# Bilateral Corneal Melting Following High-Voltage Electrical Injury: A Case Report and Review

**DOI:** 10.22336/rjo.2026.61

**Published:** 2026

**Authors:** Dharamveer Singh Choudhary, Twinkle Garg, Bhuvanesh Sukhlal Kalal

**Affiliations:** 1 Department of Ophthalmology, Swai Man Singh Medical College and Hospitals, Jaipur, Rajasthan, India; 2 Department of Pharmacology and Nutritional Sciences, College of Medicine, University of Kentucky, Lexington, Kentucky, USA

**Keywords:** electrical injury, corneal melting, amniotic membrane transplantation, retinal manifestations, ocular trauma, vision loss, AC = Alternating current, AMT = Amniotic membrane transplantation, B-scan = B-scan ultrasonography, DC = Direct current, ERG = Electroretinography, MMPs = Matrix metalloproteinases, NSAID = Nonsteroidal anti-inflammatory drug, OCT = Optical coherence tomography

## Abstract

Electrical injuries, particularly those involving high-voltage current, can cause extensive damage across multiple organ systems. Although ocular involvement is rare, the eye may develop severe, vision-threatening anterior and posterior segment complications. This report describes an unusual case of bilateral corneal melting following a high-voltage domestic electrical injury in the absence of direct ocular contact or periocular burns. A 65-year-old female presented ten days after injury with progressive bilateral visual loss, photophobia, tearing, and ocular pain. Slit-lamp examination revealed diffuse corneal edema, Descemet’s membrane folds, stromal necrosis, and large epithelial defects in both eyes. Fundus visualization was initially obscured. A diagnosis of bilateral corneal melting secondary to electrical trauma was made. The patient was started on systemic corticosteroids, topical fortified antibiotics, and lubricating agents. Despite partial clinical improvement, persistent epithelial defects necessitated bilateral amniotic membrane transplantation. Postoperatively, the ocular surface stabilized, with gradual epithelial healing and symptom resolution. By day 120, bilateral central corneal opacities with superficial neovascularization were observed. Fundus examination, possible after corneal clearing, revealed signs of age-related macular degeneration and mild optic disc pallor. Visual acuity remained at light perception in both eyes due to posterior segment pathology. This case underscores the importance of recognizing rare anterior segment complications such as sterile keratolysis in patients with electrical injuries, even when no facial burns are present. Corneal melting in this context likely results from a combination of ischemia, inflammatory enzyme activity, and neurotrophic dysfunction. Early medical intervention with corticosteroids, combined with surgical management using amniotic membrane grafting, is crucial to preserve anatomical integrity and prevent perforation. Long-term follow-up is critical to identify delayed complications, support ocular healing, and assess posterior segment status. This case contributes to the limited literature on electrical injuries with bilateral corneal involvement.

## Introduction

Electrical injuries are a complex form of trauma capable of causing widespread, multi-organ damage. The pathophysiology involves both thermal and non-thermal mechanisms, including tissue vaporization, protein coagulation, ischemia from vascular compromise, and electroporation-induced cellular injury [1,2]. The extent of systemic involvement often depends on voltage, current type (AC or DC), exposure duration, and the current’s route through the body. High-voltage injuries, typically defined as exposures exceeding 1,000 volts, pose a particularly high risk for profound tissue damage and long-term morbidity [3].

Because of their delicate structure and exposed anterior surface, the eyes are especially vulnerable to damage from electrical trauma [4]. Although ocular complications following electrocution are relatively uncommon compared to neurological or cardiac manifestations, they can involve virtually any ocular structure, ranging from the adnexa to the retina and optic nerve [5]. These complications may present acutely or manifest weeks to months after the initial injury, which can delay diagnosis and treatment [6].

Reported ophthalmic sequelae span a wide spectrum. Anterior segment findings may include eyelid burns, conjunctival hyperemia, keratitis, anterior uveitis, iris atrophy, and most notably, cataract formation [7,8]. Cataracts, especially anterior subcapsular opacities, are well-documented consequences of high-voltage electrical exposure and lightning strikes, often attributed to heat conduction through the lens or oxidative stress-induced protein denaturation [9,10]. Posterior segment complications, though less common, can include macular holes, retinal edema, retinal hemorrhages, retinal vascular occlusions, and optic neuropathy [11]. These changes are believed to result from vascular spasm, ischemia, or inflammatory damage to retinal and optic nerve tissues.

Corneal melting, also called sterile keratolysis, is a rare but potentially devastating complication of ocular surface disease [12]. It is defined by progressive stromal degradation leading to corneal thinning, ulceration, and potential perforation. The pathogenesis involves an imbalance between matrix metalloproteinases and their tissue inhibitors, typically precipitated by inflammation, ischemia, or neurotrophic dysfunction [5,13]. Corneal melt is more commonly associated with autoimmune diseases such as rheumatoid arthritis, post-infectious keratitis, ocular surface burns, or toxicity from topical medications. However, its occurrence following electrical trauma is exceedingly rare and poorly understood.

High-voltage electrical injuries may precipitate corneal melting through several mechanisms. Direct thermal injury to the corneal epithelium and stroma, vascular compromise resulting in ischemic necrosis, and cellular disruption via electroporation are all contributory factors [14-16]. Additionally, neurotrophic damage to the corneal nerves may impair healing, reduce tear production, and contribute to epithelial breakdown [17]. These effects may not be immediately apparent, with clinical signs often emerging several days or even weeks after the inciting event. This delayed presentation can further complicate the diagnosis and delay appropriate management.

Only a few cases of corneal melting after lightning strikes or electrocution have been reported in the literature, with most involving unilateral presentation [18]. This report described a focal corneal melt occurring within hours of a lightning strike and isolated cases of unilateral corneal damage after high-voltage electrocution [4,19]. To date, spontaneous bilateral corneal melting without direct ocular contact or periocular burns following domestic high-voltage electrical injury remains largely undocumented.

In this report, we present a rare and unusual case of bilateral corneal melting in a 65-year-old woman following a domestic electrical injury. We describe the clinical presentation, management course—including medical and surgical interventions—and long-term visual outcome. This case underscores the importance of early ophthalmic evaluation in patients with electrical injury, even without external ocular trauma. Furthermore, it highlights the role of timely intervention with systemic corticosteroids and amniotic membrane transplantation in stabilizing the ocular surface and preserving anatomical integrity. By contributing to the limited literature on this topic, this report also emphasizes the need for heightened clinical awareness and long-term monitoring in patients with high-voltage electrical exposure.

## Case report

### Patient history

A 65-year-old female presented to the ophthalmology outpatient department with a 10-day history of progressive vision loss, severe ocular pain, photophobia, and excessive tearing in both eyes. The onset of her symptoms coincided with a high-voltage electrical injury she sustained at home while performing routine household tasks. According to the patient, she accidentally came into contact with an exposed electrical wire, resulting in an electric shock that threw her backward. She lost consciousness for approximately five minutes following the incident and sustained a third-degree burn over the posterior aspect of her neck. There were no signs of trauma or burns to her face or eyes. After the injury, she was taken to a local healthcare provider in her village for initial treatment. However, no documentation of that treatment was available at the time of presentation. Her family brought her to our tertiary care center when symptoms worsened despite the initial treatment.

### Clinical findings

On examination at our center, her visual acuity was reduced to perception of light in both eyes, with inaccurate ray projection. There were no external signs of periocular trauma or electrical burns. Extraocular movements were full in both eyes. Intraocular pressure was 14 mm Hg bilaterally by applanation tonometry.

Slit-lamp examination revealed significant ocular surface abnormalities in both eyes (**Fig. 1A, B**). Findings included diffuse corneal edema, multiple Descemet’s membrane folds, patchy stromal necrosis, and large central epithelial defects. The conjunctiva was markedly congested and inflamed in both eyes. The anterior chambers were deep and quiet, with no signs of hypopyon or hyphema. In the right eye, the lens appeared cataractous, while the left eye was pseudophakic. Visualization of the posterior segment was not possible due to the extent of corneal edema and opacity. B-scan ultrasonography was performed in both eyes to assess the posterior segment. The scans showed normal posterior segments, with no evidence of vitreous hemorrhage, retinal detachment, or choroidal pathology.

**Fig. 1:**
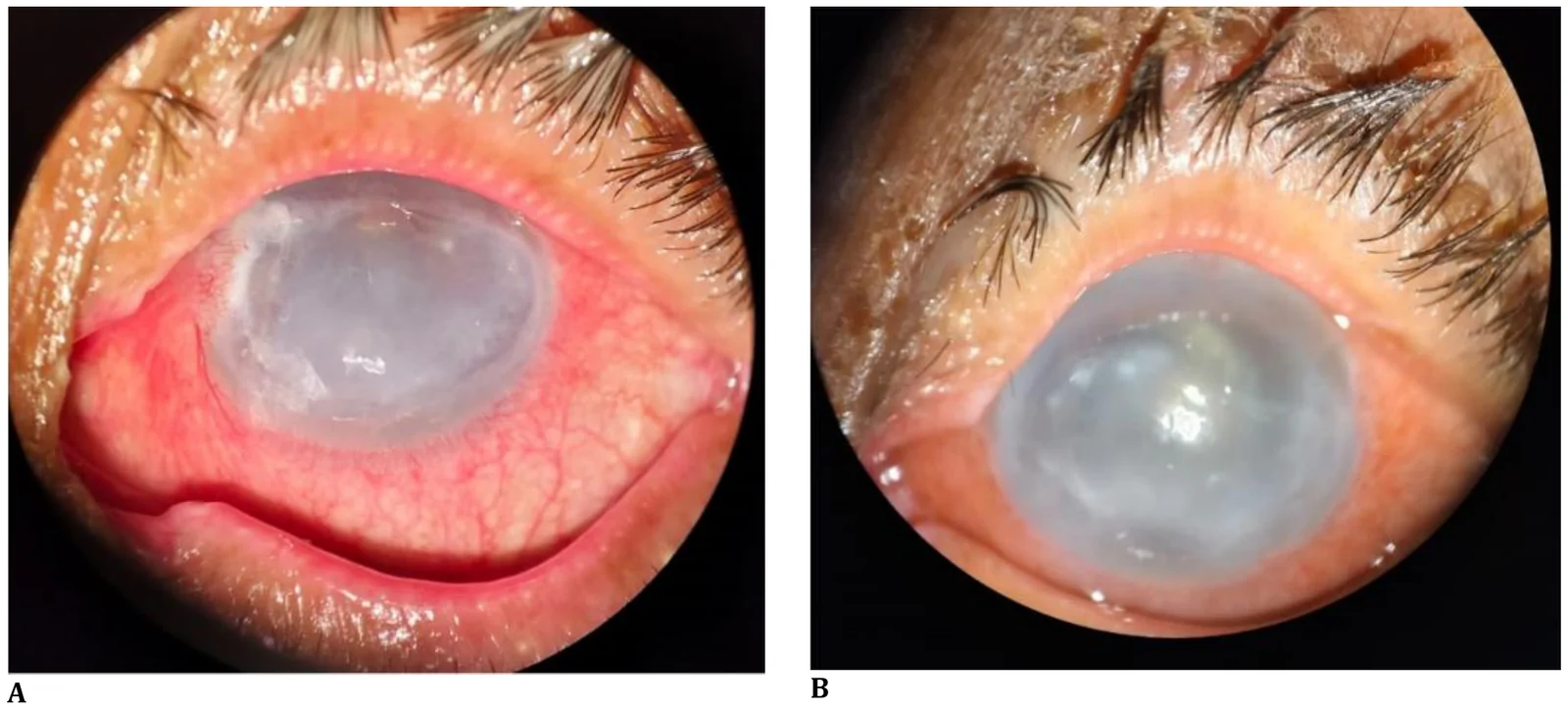
Slit-lamp photographs of both eyes at initial presentation. (A) Right eye showing diffuse corneal edema, Descemet’s membrane folds, patchy stromal necrosis, and a large central epithelial defect; (B) Left eye with similar findings, including extensive conjunctival injection and corneal surface breakdown. The posterior segment was not visualized due to corneal opacity in both eyes

### Diagnosis and management

A diagnosis of bilateral corneal melting secondary to electrical injury was made. The patient was started on oral methylprednisolone at a dose of 1 mg/kg/day, with a tapering schedule over the subsequent week. She was also prescribed topical fortified antibiotic eye drops (cefazolin and tobramycin), preservative-free lubricating drops, and close follow-up.

At follow-up on day 10, the patient showed clinical improvement (**Fig. 2A, B**). The epithelial defects had decreased in size, with a significant reduction in corneal stromal edema and conjunctival congestion. A brown nuclear cataract was now clearly visible in the right eye, and the intraocular lens in the left eye was well-positioned. However, persistent corneal haze made fundus examination inconclusive. Visual acuity improved slightly to perception of light with accurate projection of rays in both eyes. The patient also reported a noticeable reduction in photophobia, tearing, and ocular discomfort.

**Fig. 2:**
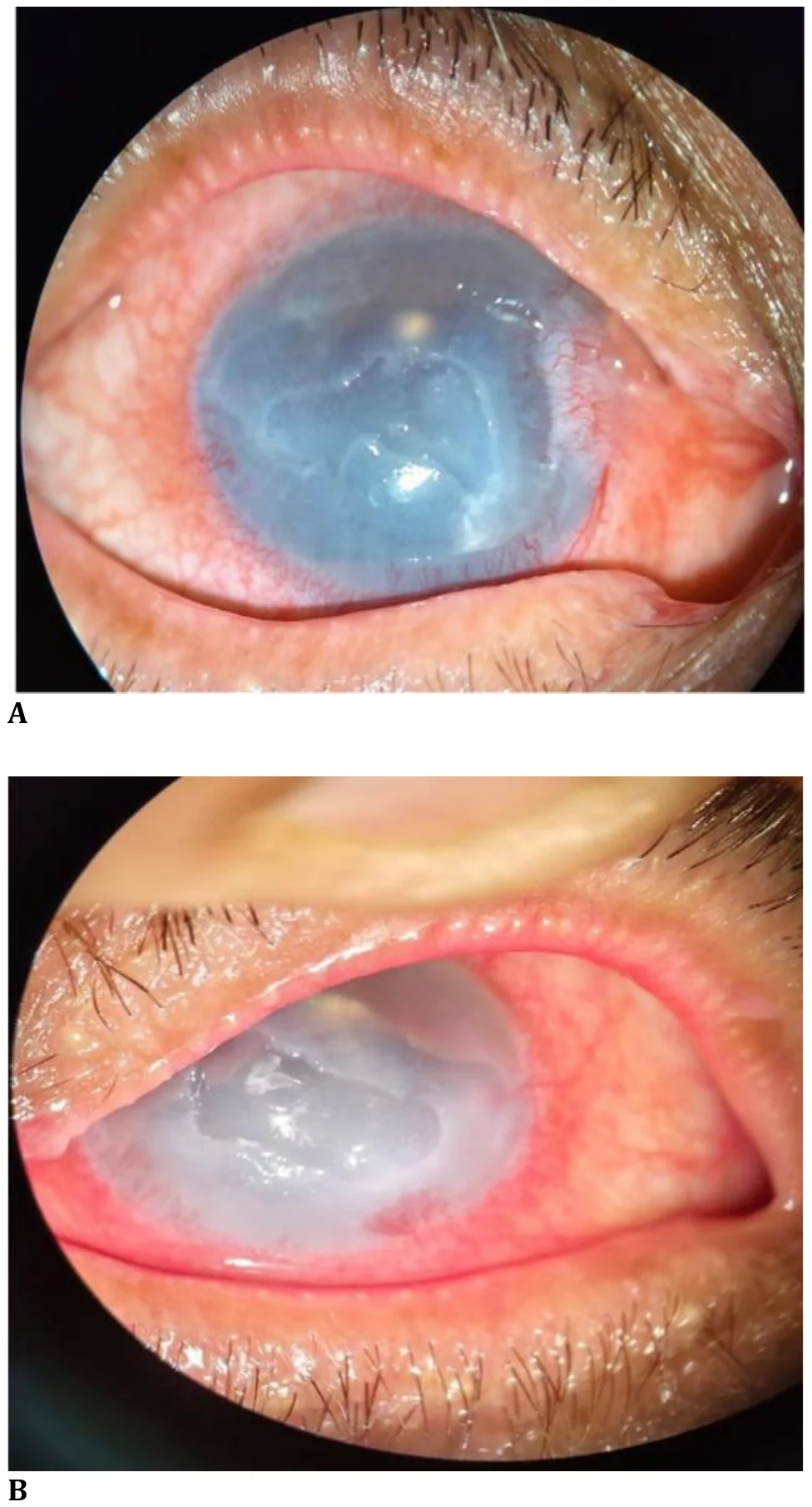
**Slit-lamp photographs on day 10 after initiation of medical therapy.** (**A**) Right eye with persistent but reduced epithelial defect, marked reduction in stromal edema, and visible brown nuclear cataract; (**B**) Left eye showing a well-positioned posterior chamber intraocular lens and decreased conjunctival congestion. Fundus remained obscured bilaterally due to corneal haze

Due to persistent epithelial defects and to prevent further stromal breakdown, the patient was admitted for surgical intervention. She underwent bilateral amniotic membrane grafting under aseptic conditions. On the first postoperative day, the amniotic membrane grafts were in place, with minimal subconjunctival hemorrhage observed in the right eye.

The patient was followed up at regular intervals on days 7, 14, 28, 60, 90, and 120. At each visit, the corneal epithelium showed progressive healing. By day 120, the patient reported no ocular pain and only mild discomfort. Slit-lamp examination revealed bilateral central corneal opacities with superficial vascularization extending from the palpebral conjunctiva (**Fig. 3A, B**). With improved corneal clarity, fundus examination became possible and showed features of age-related macular degeneration along with slight pallor of the optic disc in both eyes.

**Fig. 3:**
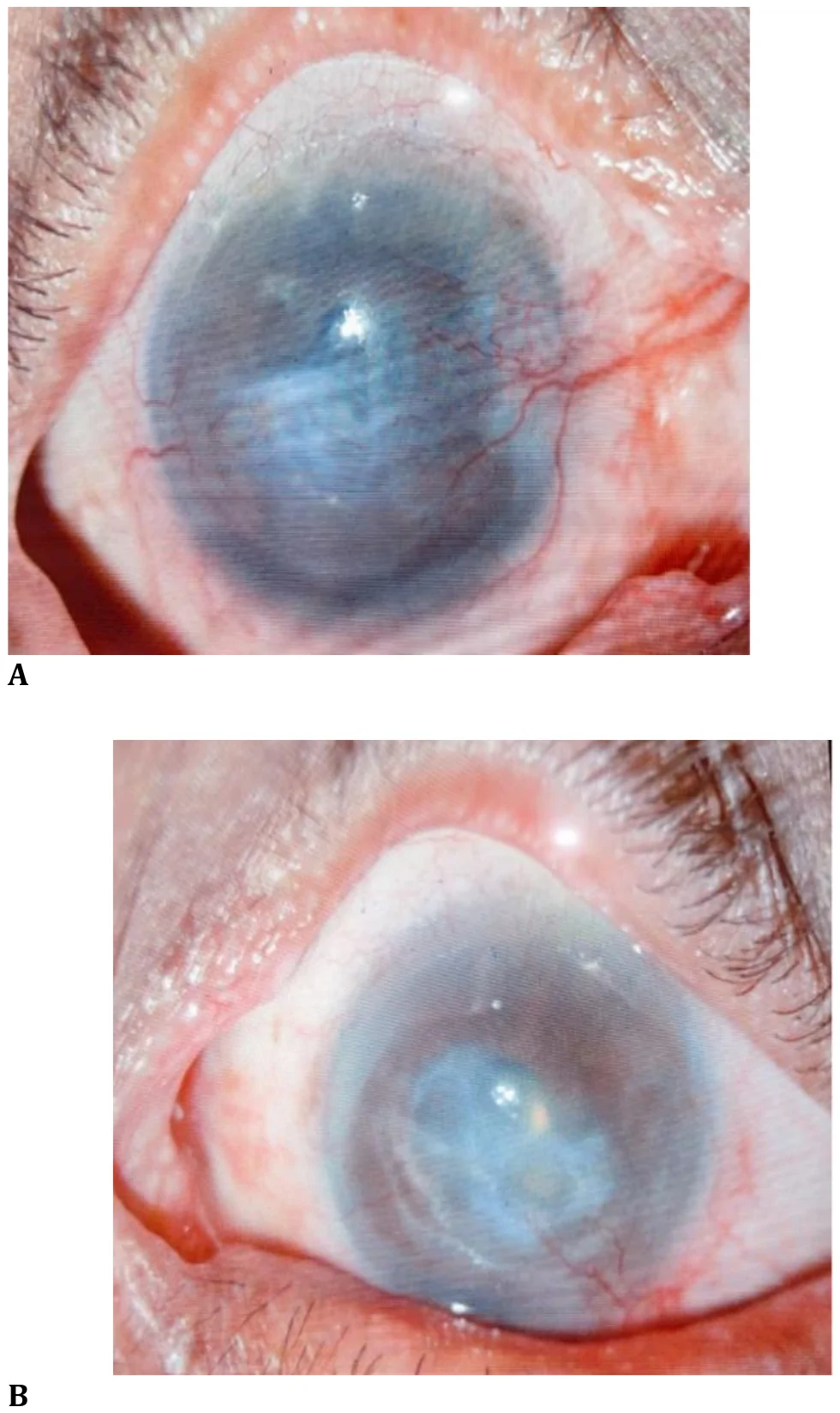
**Slit-lamp photographs on postoperative day 120 following bilateral amniotic membrane grafting.** (**A**) Right eye with central corneal opacity and superficial neovascularization extending from the palpebral conjunctiva; (**B**) The left eye shows similar corneal changes with a stable ocular surface. Patient reported resolution of pain and photophobia; fundus examination revealed age-related macular degeneration and mild optic disc pallor in both eyes

## Discussion

Electrical injuries, especially those involving high-voltage currents, can cause widespread tissue damage through direct thermal effects, vascular compromise, and inflammatory cascades. While skin and nervous system injuries are frequently documented, ocular complications, though less common, can be severe and vision-threatening [5]. The eye’s unique anatomy and physiology make it susceptible to both direct and indirect damage following electrical trauma.

Anterior segment involvement, such as corneal melting and cataract formation, is a recognized but rare consequence of electrical injury [20]. In our case, the patient developed bilateral corneal melting characterized by diffuse corneal edema, stromal necrosis, and persistent epithelial defects. Notably, there were no external burns around the eyelids or periocular region, suggesting that the corneal damage likely resulted from internal mechanisms rather than direct thermal contact.

The pathogenesis of corneal melting after electrical injury is multifactorial. One key mechanism involves ischemia due to vascular injury [21]. The cornea depends on limbal blood vessels and aqueous humor for nourishment; electrical current-induced damage to the limbal vasculature can cause ischemia, leading to stromal necrosis and impaired epithelial healing. Additionally, electric shock can trigger an intense inflammatory response, releasing matrix metalloproteinases and other proteolytic enzymes that degrade corneal stroma [22]. Oxidative stress and direct injury to corneal endothelial cells may further exacerbate tissue damage [23]. Together, these processes lead to the progressive corneal melting observed clinically.

While anterior segment complications dominate the clinical picture, posterior segment involvement must not be overlooked. Retinal manifestations after electrical injury, although rare, have been reported, including macular holes, retinal edema, hemorrhages, vascular occlusions, and retinal detachment [**7,24- 29**]. These arise primarily from thermal coagulation, vascular spasm, ischemia, and inflammatory damage (**Table 1**). Optic nerve involvement, such as pallor and atrophy, can result from ischemic injury or retrograde degeneration due to disrupted axonal transport.

**Table 1:** Retinal Manifestations Following Electrical Injury

Retinal Manifestation	Description	References
Macular Hole	Increased retinal thickness and retinal atrophy progression	[24]
Retinal Edema and Pigmentary Changes	Pigmentary changes surrounding the optic disc	[25]
Retinal Hemorrhage	Elevated subretinal haemorrhage at the macula	[26]
Retinal Detachment	Non-granulomatous anterior uveitis with nuclear cataract and total retinal detachment	[7]
Retinal Vascular Occlusion	Unilateral paracentral acute middle maculopathy and nonischemic central retinal vein occlusion	[27]
Chorioretinal Atrophy	Retinal atrophy surrounding the optic nerve	[28]
Macular cysts	Bilateral maculopathy and cataracts.	[29]

In our patient, corneal opacities initially hindered fundus visualization. Still, subsequent examination revealed age-related macular degeneration and optic disc pallor, suggesting some degree of posterior segment compromise. These findings likely contributed to the limited visual recovery despite successful treatment of the corneal pathology.

Management of ocular electrical injuries is challenging and requires a multidisciplinary approach. Prompt initiation of systemic corticosteroids is crucial to suppress inflammation and prevent further tissue degradation. Topical fortified antibiotics reduce the risk of secondary infection, which can complicate corneal ulcers. Supportive measures such as preservative-free lubricants help maintain ocular surface integrity. When medical therapy alone is insufficient, surgical interventions like amniotic membrane transplantation (AMT) play a vital role in promoting epithelial healing and restoring corneal stability, as demonstrated in this case [30].

Given the potential for delayed complications involving both anterior and posterior segments, careful long-term follow-up with regular slit-lamp and fundus examinations is imperative. Ancillary investigations such as optical coherence tomography (OCT) and electroretinography (ERG) may provide valuable insights into retinal structure and function, guiding prognosis and further management.

This case highlights several important clinical lessons. First, ocular involvement in electrical injuries may be under-recognized, especially in the absence of external facial burns. Second, corneal melting can progress rapidly if not addressed promptly. Third, early recognition and combined medical and surgical interventions, including corticosteroids and AMT, can help preserve ocular integrity and reduce complications. Lastly, close and prolonged follow-up is essential to monitor healing and manage delayed-onset complications such as scarring, neovascularization, and posterior segment changes.

## Conclusion

Bilateral corneal melting following high-voltage electrical injury is an exceptionally rare and vision-threatening complication that can occur even in the absence of direct ocular trauma or facial burns. This case underscores the need for a high index of suspicion and thorough ophthalmic assessment in all patients presenting after electrical shock, regardless of external ocular findings. Early diagnosis, prompt initiation of systemic and topical anti-inflammatory therapy, and timely surgical intervention with amniotic membrane transplantation are critical to preserve corneal integrity and optimize visual outcomes. Long-term follow-up remains essential to manage delayed complications and to support functional visual rehabilitation. This case also highlights the importance of interdisciplinary collaboration in managing complex electrical injuries with ocular involvement.
